# Supplementary figures and images for: Identification of a Putative CodY Regulon in the Gram-Negative Phylum Synergistetes
Source: Int J Mol Sci. 2022 Jul 18;23(14):7911. doi: 10.3390/ijms23147911 (PMC9318921; doi:10.3390/ijms23147911)

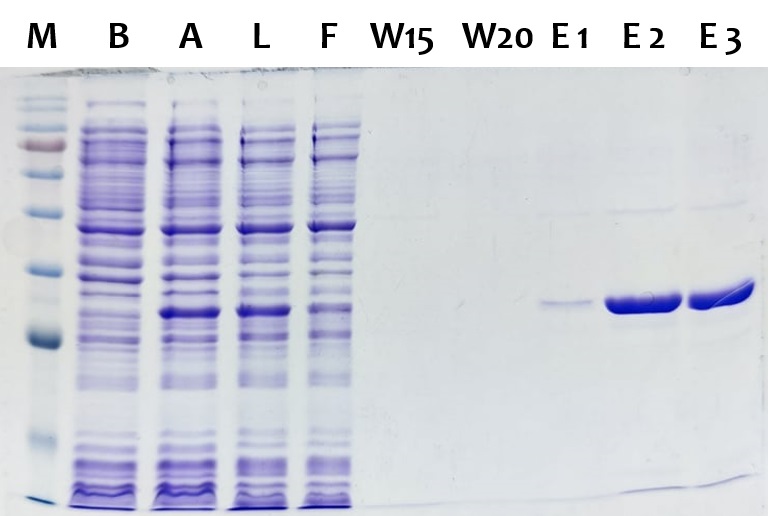

Supplement: Supplementary file 1 [file ijms-23-07911-s001.zip › ijms-1797246-Figure S1.jpg]
